# A topological hypothesis for atrial fibrilllation, atrial flutter and focal atrial tachycardia: comparison and contrast with Kosterlitz-Thouless physics

**DOI:** 10.3389/fnetp.2025.1710567

**Published:** 2026-01-08

**Authors:** Anand Narayan Ganesan, Pawel Kuklik, Stanley Nattel

**Affiliations:** 1 College of Medicine and Public Health, Flinders University, Adelaide, SA, Australia; 2 Universite de Montreal, Montreal, QC, Canada

**Keywords:** atrial fibrillation, atrial flutter, Kosterlitz-Thouless, topology, atrial tachycardia

## Abstract

While the role of topology is established in active matter systems, its importance in cardiac electrophysiology, particularly concerning common arrhythmias, warrants further emphasis. Atrial fibrillation (AF), atrial flutter (AFL), and focal atrial tachycardia (FAT) are the most prevalent arrhythmias impacting human health. This article proposes a framework conceptualizing these atrial rhythm disturbances through the lens of topological states and phase transitions, drawing inspiration from the Kosterlitz-Thouless (KT) transition. Central to this framework is the hypothesis that distinct arrhythmia patterns emerge as discrete topological states constrained by the fundamental requirement that the net topological charge (associated with electrical phase singularities or vortices) must sum to zero across the atrial tissue. Within this constrained topological perspective, AF, characterised by disorganised activity, is likened to the KT unbound vortex state, dominated by disorder with repetitive vortex regeneration and an exponential decay in spatial correlation. In contrast, AFL, with its organized regularity, resembles the KT bound vortex state, where vortex-antivortex pairs result in ordered activity. Finally, FAT and Sinus Rhythm are characterized as topologically vortex-free states exhibiting ordered planar wave conduction. Importantly, while the resulting topological states show clear analogies, the specific biophysical mechanisms driving vortex defect formation, interaction, and unbinding in cardiac tissue likely differ significantly from the thermal free-energy considerations governing the classic KT transition. This viewpoint frames the transition between arrhythmias as a change in the topological organization of atrial electrical activity, governed by charge conservation principles and cardiac-specific dynamics. This perspective may offer novel diagnostic and therapeutic avenues applicable to human cardiac mapping procedures.

## Introduction

Atrial fibrillation (AF), atrial flutter (AFL), and focal atrial tachycardia (FAT) are the most common human arrhythmias interrupting normal sinus rhythm (SR). Despite extensive research and numerous advancements in understanding, AF remains a complex and controversial disorder (Nattel, 2002). At present, however, the definitive mechanism of AF remains elusive (Callans, 2020; Nattel and Dobrev, 2017; Nattel et al., 2017; Roney et al., 2020; Heijman et al., 2021; Jalife, 2011). Emerging evidence suggests that continuous regeneration of vortices is an important mechanism sustaining AF (Dharmaprani et al., 2019; Dharmaprani et al., 2022; Dharmaprani et al., 2021; Jenkins et al., 2023; Jenkins et al., 2022; Quah et al., 2021). AFL and AT, on the other hand, are considered comparatively resolved both in terms of its mechanism and treatment (Callans, 2020; Saoudi et al., 2001). Typical AFL is conventionally described as a fixed circuit around the tricuspid annulus (Saoudi et al., 2001; Arribas et al., 1997; Cosio, 2017; Cosio et al., 1996a; Cosio et al., 1996b; Roberts-Thomson et al., 2006; Issa et al., 2009). Atypical flutter, which accounts for 10% of AFL cases, presents a more complex clinical challenge with diverse and intricate electrophysiological patterns that complicate diagnosis and treatment (Jais et al., 2009; Jaïs et al., 2000; Kalman et al., 1997). FAT, which accounts for around ∼10% of supraventricular arrhythmia, is the third primary atrial arrhythmia, characterised by abnormal automatic or micro-re-entrant activity driving organised activity in the atrial chambers (Callans, 2020; Roberts-Thomson et al., 2006; Issa et al., 2009).

An intriguing aspect of the clinical care of atrial arrhythmia patients is the complex overlap between AF, AFL, and FAT (Roithinger et al., 1997). In patients, although the arrhythmias are considered as distinct entities, there is evidence of frequent transitions between AF, AFL, and FAT, with these entities often coexisting at different times in the same patients (Roithinger et al., 1997). Contemporary clinical data suggests that AF will develop in at least 77% of AFL patients (Attanasio et al., 2024), and pulmonary vein isolation, the modern approach to treatment of AF (Haissaguerre et al., 1998), is surprisingly clinically effective in preventing AFL (Gupta et al., 2023).

To date, conventional clinical approaches to understanding AF, AFL, and FAT generally consider these arrhythmias as distinct disease processes with distinct anatomical and physiological underpinnings (Callans, 2020; Saoudi et al., 2001; Issa et al., 2009). However, recent clinical evidence suggests that topology of wave propagation may play a role in defining the phenotypic differences between these arrhythmias. Emerging data suggest that: (i) AFL may considered as a bound vortex state, with paired chiral and counter-chiral circuits fundamental to arrhythmogenesis (Duytschaever et al., 2024; Vandersickel et al., 2024; Santucci et al., 2024), (ii) AF may considered as the consequence of repetitive regeneration of unstable topological vortices (Dharmaprani et al., 2021; Jenkins et al., 2023; Jenkins et al., 2022; Dharmaprani et al., 2021; Dharmaprani et al., 2019; Dharmaprani et al., 2024), related to concepts of scroll wave turbulence (Christoph et al., 2018; Fenton et al., 2002; Karma, 1994; Kuklik et al., 2010; Panfilov and Pertsov, 2001; Panfilov, 1998; Ten Tusscher and Panfilov, 2003; Ten Tusscher and Panfilov, 2006; Clayton, 2008; Yang et al., 2003; Qu et al., 2000). Collectively, these observations are the basis for the hypothesis proposed in this contribution that the inter-relationship AF between and AFL could potentially be considered as alternative topological phases, with possible useful parallels to defect unbinding topological phase transitions observed in Kosterlitz-Thouless physics (Bowick et al., 2022; Shankar et al., 2022).

**FIGURE 1:**
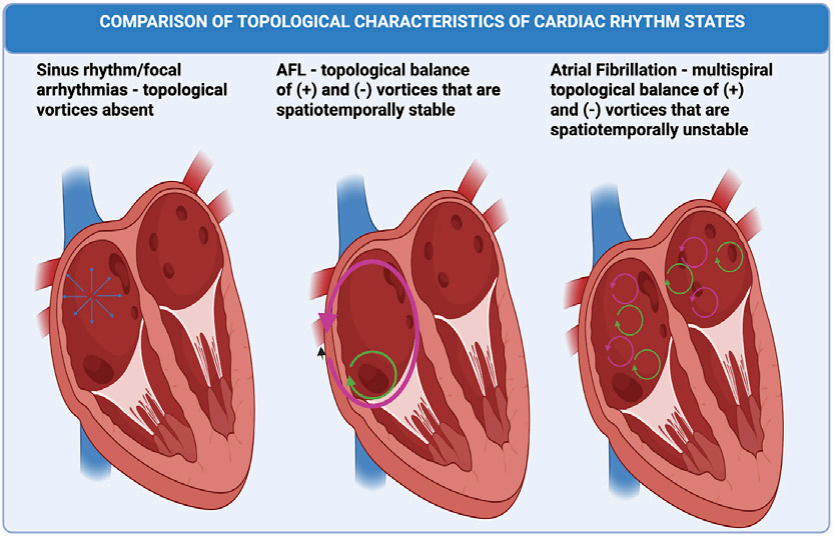
Overview of the Topological Phase Hypothesis for Atrial Arrhythmias. Conceptual illustration of the proposed topological states for common atrial arrhythmias. Sinus Rhythm (SR) and Focal Atrial Tachycardia (FAT) are depicted as vortex-free states with planar wave propagation and high spatial correlation. Atrial Flutter (AFL) is represented as a bound vortex state, characterized by paired, counter-chiral vortices (+1 pink/−1 green) leading to quasi-long-range order. Atrial Fibrillation (AF) is shown as an unbound vortex state, with chaotically regenerating and annihilating vortex pairs, resulting in topological disorder and an exponential decay in spatial correlation.

We propose a topological hypothesis that views AF, AFL, and FAT as separate, but related, topological states from SR, drawing inspiration from the principles of topological phase transitions. Our objective is to analyze the topological features of AF, AFL, FAT comparatively, using Kosterlitz-Thouless (KT) transition principles (Kosterlitz, 2017; Kosterlitz, 2016) to highlight similarities and differences and deepen our understanding of these arrhythmias. We also include SR in our formulation as the reference ordered state against which these arrhythmias are compared. Figure 1 illustrates our proposed topological framework, showing how AF, AFL, AT and SR can be characterized as distinct topological states based on the presence and organization of phase singularities (vortices). We are careful to specifically avoid claiming that the transitions between these dysrhythmias are an example of a KT-type transition. However, the aim of this contribution is to propose a topological hypothesis of AF, AFL, and FAT that regards these arrhythmias as separate distinct but related topological states, inspired by the principles of Kosterlitz-Thouless (KT) physics (Kosterlitz, 2017; Kosterlitz, 2016).

To elaborate on this topological hypothesis, the following sections delve into several key areas. We begin by providing an overview of the relevant principles of Kosterlitz-Thouless (KT) physics, which serves as a prototype topological phase transition. Building on this, we compare and contrast the necessary principles of KT-like physics relevant to characterizing AF, AFL, and FAT/SR (Figure 1) as alternative topological states. We then examine the implications of the thermodynamic limit for these cardiac topological transitions occurring in a nonequilibrium state, specifically identifying how their defect unbinding mechanism differs fundamentally from equilibrium cases. We conclude by discussing potential predictions about the nature of AF, AFL, and FAT derived from this conceptualization, and present possible use cases for this approach to improve clinical and mechanistic understanding of these disorders.

The intention of our paper is to propose that *topological phase transitions* provide a useful point of comparison for understanding the nature and transition of these arrhythmias as topologically alternative states. Specifically, we propose that AF can be understood as an unbound vortex state, atrial flutter (AFL) corresponds to a bound vortex state, while FAT is a dysrhythmia with absent vortices (Figure 1). SR as the reference ordered state is topologically comparable to FAT by the absence of vortices (Figure 1). By consideration of AF, AFL FAT and SR as alternative topological states, we hope to potentially facilitate understanding of the processes underlying the frequent transitions between these arrhythmias and sinus rhythm and hence pave the way for new treatment approaches.

In the following sections, we will: (i) provide an overview of the relevant principles of Kosterlitz Thouless (KT) physics as a prototype topological phase transition; (ii) compare and contrast the necessary principles of KT-like physics that might allow the disorders of AF, AFL, and FAT to characterise these as alternative topological states, (iii) discuss the issue of the thermodynamic limit, and why a topological approach to AF, AFL, and FAT might occur in the heart as a nonequilibrium state, identifying key differences in the underlying nature of the mechanism of defect unbinding; (iv) discuss potential predictions about the nature of AF, AFL, and FAT that would flow from conceptualisations of these dysrhythmias as alternative topological phases, and evidence supporting this concept in clinical and mechanistic studies.

### Overview of Kosterlitz-Thouless physics

Kosterlitz-Thouless (KT) physics emerged from the study of phase transitions in two-dimensional systems, leading to the Nobel Prize for J.Michael Kosterlitz and David J. Thouless in 2016 (Kosterlitz, 2017; Kosterlitz, 2016). Berenzinskii made similar arguments contemporaneously in the Soviet literature (Berezinskii, 1971). It describes a unique kind of phase transition driven by the binding and unbinding of vortex-antivortex pairs. In a KT transition, the system undergoes a shift from a low-temperature phase, where vortex-antivortex pairs are tightly bound, to a high-temperature phase, characterized by free, unbound vortices. This transition is governed by the interplay of free energy, configurational entropy, and the underlying topological characteristics of the system.

Topological vortices play a crucial role in Kosterlitz-Thouless (KT) physics, particularly in two-dimensional systems where they act as the primary determinants of topological phase. These vortices arise due to the unique nature of the phase angle in such systems. In the context of the XY model, which was the basis of the original theoretical work of Kosterlitz and Thouless (Kosterlitz and Thouless, 1973), spins are represented by vectors in a plane, characterized by an angle ϕ (ϕ relative to a reference direction). In the XY model, spins are continuously valued, each represented by an angle ϕ in the range of 0 to 2π, lying on a circle. A vortex configuration occurs when the phase angle ϕ wraps around a topological defect (effectively a “hole”) by an integer multiple of 2*π*, so that a closed loop traverses around a central point (Equation 1).

$$\upsilon =\frac{1}{2\pi }\oint \nabla \phi dl$$ (1)

The occurrence of topological vortices in Kosterlitz-Thouless physics can be understood through the lens of homotopy theory, specifically the first homotopy group, π_1_ (Mouc et al., 2018). In a two-dimensional system, the order parameter space, which in the case of the XY model is the circle S1, plays a critical role (Mouc et al., 2018). The first homotopy group π_1_(S1) consists of all possible loops in S1 that can be continuously deformed into one another, each loop corresponding to an integer winding number (Mouc et al., 2018). These winding numbers reflect how many times the phase angle ϕ traverses a closed loop (Chinn and Steenrod, 1966). A vortex corresponds to a nontrivial element of this homotopy group, where the phase angle changes by 2π*n* (with n being a nonzero integer) around a closed path (Mouc et al., 2018). This topological quantization ensures that vortices are stable, discrete entities that cannot be removed by continuous transformations. An additional point that must be understood is that ν must be zero for closed paths not enclosing phase singularities and that the sum of ν over the whole system must be zero (Bray and Wikswo, 2002; Pertsov et al., 2000; DeTal et al., 2022).

In Kosterlitz-Thouless (In KT) physics, topological phase is understood through free energy considerations, reflecting the intrinsic competition between energy and entropy for vortex configurations. The energy required for forming an individual vortex, $E_{v}$ , is calculated as Equation 2:

$$E_{v}=E_{c}+J\pi n^{2}\ln\left(\frac{L}{a}\right)$$ (2)

In this formula, $E_{v}$ represents the total vortex energy, $E_{c}$ is the energy to form the core, J is the interaction strength constant, n is the winding number, L is the system size, and a is the lattice spacing. As shown, $E_{v}$ scales logarithmically with the system size L and quadratically with the winding number n . This dependence on system size highlights the substantial energy barrier to forming a vortex, while the n ^*2*^ scaling intrinsically favours vortices with the minimal winding number, n=1 .

In the KT bound vortex phase, topological vortices typically occur in pairs, a phenomenon that can be understood through energetic principles. The equation for a pair of vortices is Equation 3:

$$\beta E=\beta E_{cores}+J\pi {\left(n_{1}+n_{2}\right)}^{2}\ln\left(\frac{L}{a}\right)+J2\pi n_{1}n_{2}\ln\left(\frac{a}{r}\right)$$ (3)

Where β=1/kT , where k is the Boltzmann constant, $E_{cores}$ is the energy to form the core of each of the vortices, $n_{1}\&\;n_{2}$ are the chirality of the two vortices, L is the system size, a is the lattice space, and r is the distance between the two vortex cores. A key characteristic of the KT bound vortex phase is the prevalence of vortex-antivortex pairs. Energetic considerations favour the formation of such pairs, especially those with winding numbers $n_{1}\&\;n_{2}\;1\;and-1$ , as their energy is lowest at minimal separation. At low temperatures (or high connectivity), these pairs remain tightly bound, ensuring quasi-long-range order throughout the system. However, upon increasing temperature towards the KT transition point, thermal fluctuations induce pair dissociation, resulting in a proliferation of free vortices.

The KT transition point occurs at the following temperature, by an argument based on the minimisation of configurational entropy (Equation 4):

$$F=\left(J\pi -2T\right)\ln\left(\frac{L}{a}\right)T_{c}=\frac{\pi }{2}J$$ (4)

The reason for this transition is that as temperature rises, the entropy gain from configurational entropy due to unbound vortices lowers the effective free energy of creating new vortices, driving the system into a disordered state with repetitive creation and destruction of vortices (Cheung, 2011).

### Alternative topological phases are characterized by differences in spatial correlation

Distinct topological phases manifest through qualitatively different behaviors in their spatial correlation functions. Spatial correlation is typically measured via the following correlation function (Equation 5):

$$G\left(r\right)=\langle e^{i\left[\phi \left(x\right)-\phi \left(x+r\right)\right]}\rangle$$ (5)

where G(r) represents the spatial correlation function, ϕ(x) represents phase at position x, and *ϕ(x + r)* represents phase at a distance r away. The bound vortex phase is characterised by paired positively chiral and negatively chiral vortices. In this phase, known at the KT phase, the system adopts a configuration of quasi-long-range order (QLRO). (Cheung, 2011) In QLRO, spatial correlations decay very slowly, according to an algebraic function of distance (Bowick et al., 2022) (Equation 6).

$$G\left(r\right)∼r^{-\eta }$$ (6)

where η is a correlation exponent that characterizes the rate of algebraic decay.

**FIGURE 2:**
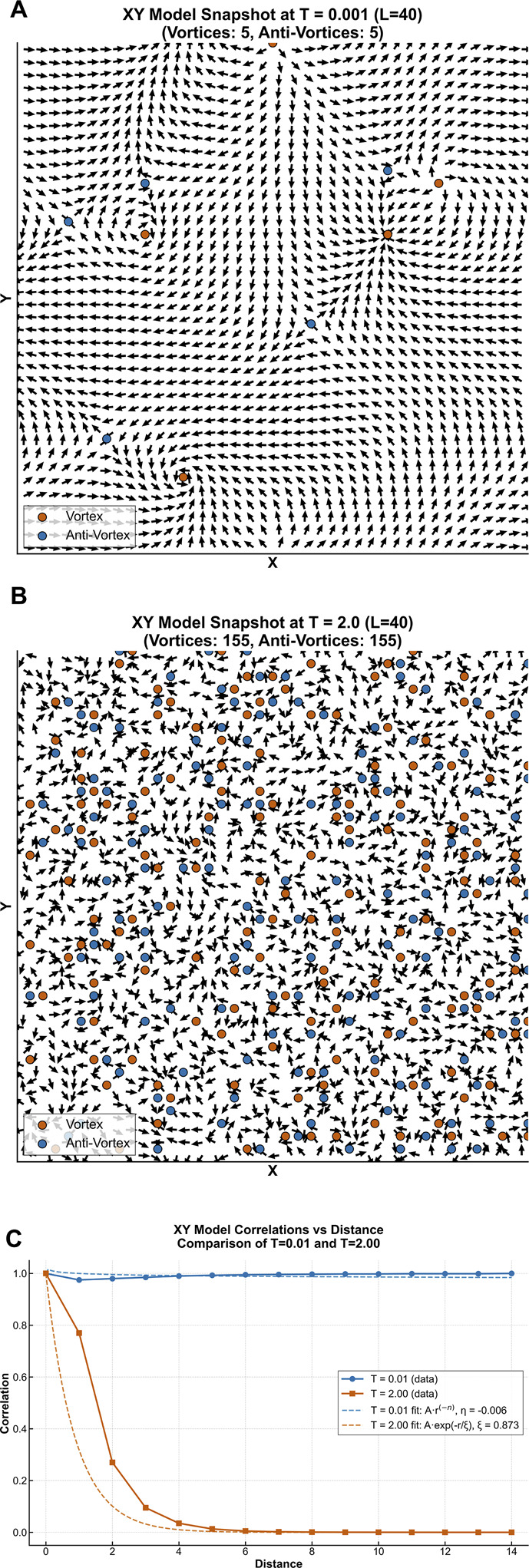
Comparison of Vortex States and Spatial Correlation in the 2D XY Model (Kosterlitz-Thouless Physics). Representative configurations and spatial correlation functions for the two-dimensional XY model demonstrating Kosterlitz-Thouless physics principles. **(A)** Low-temperature bound vortex phase (T < T_c): Stable vortex-antivortex pairs (red = +1, blue = −1) with quasi-long-range order exhibiting algebraic correlation decay G(r)∼r^(-η) where η < 0.25. **(B)** High-temperature unbound vortex phase (T>T_c): Proliferation of free, unbound vortices with exponential correlation decay G(r)∼e^(-r/ξ), where ξ is the correlation length. **(C)** Quantitative comparison: Plot showing the distinct scaling behaviors - slow algebraic decay (bound phase) versus rapid exponential decay (unbound phase), analogous to the proposed AFL and AF states respectively.

The alternative topological state, the unbound vortex state, is characterised by repetitive regeneration of vortices. In this state, the spatial correlation function decays rapidly (exponentially) with distance (Cheung, 2011) (Equation 7).

$$G\left(r\right)∼e^{\frac{-r}{\varepsilon }}$$ (7)

where ε (epsilon) is the correlation length, representing the characteristic distance over which spatial correlations decay to 1/e ≈ 37% of their initial value. In disordered states with proliferating topological defects, ε is typically much smaller than the system size (ε << L), resulting in rapid loss of spatial coherence. A key point is that this state is favoured by larger system size, leading to increased configurational entropy (essentially because there are more potential locations for the vortices to be located in the system), allowing this disordered state to become energetically favoured. Figure 2 shows example configuration and spatial correlations for the 2D XY model in the KT bound vortex (Figure 2A) and unbound vortex states (Figure 2B). The key points that can be determined are that: (i) in the low temperature phase, the system is relatively ordered with a slow algebraic decay in spatial correlation; and (ii) in the high temperature phase, the system is disordered with an exponential decay in spatial correlation (Figure 2C). For atrial fibrillation, clinical studies have reported exponential decay in correlation, indicating that electrical activity becomes uncorrelated over distances spanning only a few electrode spacings (Dharmaprani et al., 2024).

### Necessary conditions for topological phase transitions

KT typological physics has found applications in various fields, including superfluid films, thin superconductors, and liquid crystals (World Scientific Europe, 2012). In each of these systems, these materials undergo topological phase transitions from bound vortex states to disordered unbound vortex states. For topological type physics to apply, the key condition is that the system must exhibit U (1) symmetry (Bowick et al., 2022), allowing for the existence of vortices as topological defects. The key condition of KT systems is that there is U (1) symmetry of the phase field ϕ, a condition of the XY model in the original ideas of Michael Kosterlitz and David Thouless.

### KT-like unbinding can occur in non-equilibrium topological active matter systems

Historically, binding-unbinding transition originally proposed in KT physics was defined primarily for equilibrium systems operating at or near the thermodynamic limit (Kosterlitz, 2017; Kosterlitz, 2016; Kosterlitz and Thouless, 1973). Recently, however, there has been growing recognition that KT-like transitions can occur in non-equilibrium systems (Bowick et al., 2022; Shankar et al., 2022). The most important and relevant transition in this regard is in the context of topological active matter systems (Shankar et al., 2018). In these systems, KT-like transitions of bound defects to unbound defects drive the transition from the nematic to the isotropic phase (Shankar et al., 2018). Unlike in original KT physics, these topological transitions may occur despite the systems being active, biological nonequilibrium systems (Bowick et al., 2022).

A key distinction in nonequilibrium systems, compared to KT physics, is the source of the fluctuations causing defect unbinding. Theoretical work, such as studies employing a complex-Ginzburg-Landau reaction-diffusion model, suggests the possibility of KT-like defect unbinding (Granzow and Riecke, 2001). These studies propose that intrinsic chaotic dynamics within the nonequilibrium system generate the necessary fluctuation spectrum to drive topological transitions between states, acting as a direct analogue to the role of thermal noise in equilibrium unbinding processes (Granzow and Riecke, 2001). In essence it has been proposed that deterministic chaos may provide the necessary randomness and fluctuation spectrum to induce transitions between disordered and ordered states, effectively serving as a nonequilibrium analogue to thermal noise in equilibrium systems (Kosterlitz, 2016).

### Implications and significance of the topological phase transitional paradigm

Topological phase properties are distinct and unique in comparison to conventional phase transitions. These are states of matter fundamentally characterized by non-local, topological properties rather than by the pattern of symmetry breaking that defines conventional phases. They represent a fundamentally different way matter can organize itself, often involving intricate patterns of long-range order among its constituents.

Topological phases exhibit at least two relevant characteristics:

⁃ *Robustness*: The defining topological properties of KT-type systems are inherently stable against local perturbations, such as impurities, defects, or continuous variations in system parameters. This remarkable stability arises because topological invariants are typically integers and cannot change smoothly; they can only jump discontinuously when the system undergoes a topological phase transition, usually associated with the closing and reopening of a free energy gap.
⁃ *Non-local global Order*: Unlike the local order of conventional phases, topological order is non-local, encoded in the global structure of patterns across the entire system. It cannot be detected by purely local measurements.

### Hypothesis: the AFL to AF conversion is a topological phase transition

This perspective hypothesizes that Atrial Flutter (AFL), Atrial Fibrillation (AF), and Focal Atrial Tachycardia (FAT) represent distinct topological states of cardiac electrical activity. We propose that transitions between these states could be viewed as topological phase transitions. Drawing an analogy to Kosterlitz-Thouless (KT) physics, we suggest AFL resembles a bound vortex state, while AF corresponds to an unbound state with repetitive vortex regeneration. FAT, similar to sinus rhythm, is proposed as a state lacking vortex singularities.

### Topological characterisation of atrial rhythm states

In this paper, it is proposed that these topological principles allow the 4 discrete states of the atrial rhythm to be defined, predicting the following properties:

- Sinus rhythm – planar wave propagation across the bi-atrial surface, with origin of conduction at the sino-atrial node. In this scenario, topological charge is 0 at all points in the atrium, including around fixed boundaries such as valve rings, veins, and scars.
- Focal atrial rhythms (especially FAT) – planar wave conduction, with origin outside the sino-atrial node. In this scenario, topological charge is 0 at all points in the atrium, including around fixed boundaries such as in the sinus rhythm case.
- AFL – topologically paired vortices, which clockwise and anti-clockwise vortices are paired (with opposite winding numbers −1/+1). Vortices may occur around fixed anatomical structures (e.g., valve rings, veins) or around discrete scars with poor/absent local wave propagation. This paired structure leads to long-range topological order.
- AF – in which vortex pairs (−1/+1) are continuously formed and annihilated, leading to disorder that is characterised by exponential decay in local spatial order.
- The sinus rhythm and FAT states would be characterised by the absence of vortices, and the presence of long-range spatial correlation.

### Mapping cardiac dynamics to a phase field model

The origin of the topological hypothesis of AF, AFL, and FAT lies in the intrinsic properties of cardiac cycling. In cardiac tissue, the cyclic nature of the—characterized by depolarization and repolarization—can be translated into a continuous phase variable, θ, that captures the travelling wave progression of electrical activity across the myocardium (Tyson and Keener, 1988). One common approach is to derive this phase variable from the voltage signal using the Hilbert transform (Bray and Wikswo, 2002; Iyer and Gray, 2001). Specifically, the phase can be defined as Equation 8:

$$\theta \left(t\right)=\arctan\left(\frac{Η\left\{V\left(t\right)\right\}}{V\left(t\right)}\right)$$ (8)

where V (t) is the local voltage recording and $Η\left\{V\left(t\right)\right\}$ denotes its Hilbert transform. This mapping converts the oscillatory behavior of cardiac cells into a phase that ranges continuously from 0 to 2π. Once the phase field is established, it becomes possible to identify topological defects—points where the phase is undefined or discontinuous. These defects are mathematically characterized by the local topological charge, computed over a closed loop C around an individual point (Equation 9):

$$q=\frac{1}{2\pi }\oint_{C}\nabla \theta \;dl$$ (9)

The value of *q* is zero in points derived from regions with planar conduction. On the other hand, a nonzero value of q (typically ±1) signals the presence of a vortex, which in cardiac tissue represents a re-entrant circuit or spiral wave in 2-dimensions. In 3-dimensions, these point-like vortex cores are postulated to trace out continuous lines through the tissue, forming structures called vortex filaments, with related 3-D spiral structures known as scroll waves (Clayton, 2008; Pertsov et al., 2000). This formalism connects the microscopic electrical behavior of individual cells with the macroscopic phenomena observed in arrhythmias.

### Topological constraints on cardiac conduction

It is important to recognise that there are topological constraints on wave propagation. The first is that due to the intrinsically locally connected nature of the myocardial network, the phase field can be assumed to be continuously differentiable, except at singularity points. In consideration of surface conduction, it can be considered as a 2D surface that is compact and orientable (Davidsen et al., 2008; Davidsen et al., 2004). This manifold may be divided into polygons, where no edges or vertices pass through singularity points (Davidsen et al., 2004).

The overall sum of topological charge *q* across the whole phase field of myocardial surface manifold *M* is zero, i.e., Equation 10:

$$\sum_{i}q_{i}=0\;for\;all\;charges\;on\;M\ldots$$ (10)

This can be derived as follows Pertsov et al. (2000); Davidsen et al. (2004). In the manifold M without boundaries, each edge of the triangulation is an edge of two polygons. Hence, the integral sums up the change in phase angle along the edges of triangles across the phase field. However, because of the fact each edge is traversed twice in opposite directions, the net contribution of each edge is 0. Hence the sum of topological charge across the whole phase field is zero.

This theorem, known as the index theorem, has been extended to manifolds with boundaries (Davidsen et al., 2004; Arno et al., 2024a; Arno et al., 2024b). Recognising that the heart is a closed surface with holes effectively punctured by non-conducting valve rings, veins, and scars, in an extension of the index theorem, it was demonstrated that the sum of indices would again sum to zero (Davidsen et al., 2004). More specifically, the index of a hole could be defined as the integral of the curve enclosing the hole, and should, by a topological argument, be ±1 as for other singularities. However, the principle of topological charge conservation remains preserved.

Davidsen and Kapral considered a number of important scenarios in the context of active spiral wave dynamics (Davidsen et al., 2004). Topological charge conservation is preserved during the formation and destruction of phase singularities. These events typically occur in pairs, where a positive charge (+1) and negative charge (−1) are created or destroyed simultaneously. An important case, however, is the scenario in which a phase singularity (PS) annihilates with a boundary. In this case, the index of the singular point and that of the boundary both change simultaneously (collapse to zero), preserving overall topological charge conservation in the system.

The principle of topological charge preservation has been extensively considered in 3-dimensions (Pertsov et al., 2000), where it is especially pertinent in the context of a third case, scroll wave turbulence, that is considered the analogue of cardiac fibrillation. In scroll wave turbulence, Pertsov et al. demonstrated that topological charge conservation extended to both the case of free wave breaks and filaments bound to the surface of the medium (Pertsov et al., 2000). In the case of wavebreaks, the ends are considered to have topological charge ±1 and so contribute to a net charge of 0 to the overall system (Pertsov et al., 2000). An important case to consider however, is where the filament is bound to the surface of the medium. Pertsov et al. proved this case as follows:

‘Indeed, consider any bound segment which is not closed on itself. It must then connect the end of a free segment with charge +1, to the beginning of another free segment with charge −1. Since each closed 2-dimensional boundary must contain a whole number of bound segments we deduce Rule (1) [principle of topological charge conservation on the surface] (Pertsov et al., 2000).

In essence, the principles of phase continuity thus provide a set of organising principles for permitted configurations in topological materials. These can be summarised in the following rules (Gray et al., 1995; Gray et al., 1998):

- Phase lines do not intersect.
- Phase lines are joined by isophase lines to two singularities of opposite topological chirality, or a boundary (where the wave circulates with opposite chirality to attached PS).
- Phase singularities are formed and destroyed in pairs.
- The overall topological charge of the system is 0.

In this paper, it is proposed that these topological principles allow the 4 discrete states of the atrial rhythm to be defined in topological terms.

### Diagnostic considerations in determination of atrial rhythm states

The different cardiac rhythm states are thus distinguished topologically based on two criteria:

- Presence or absence of spatiotemporal order.
- Presence and extent of topological charged vortices.

The spatial organization of cardiac electrical activity can be quantitatively assessed using mean phase coherence (MPC) as a measure of phase synchronization between signal pairs (Mormann et al., 2000). MPC quantifies how consistently the phase difference between two signals remains consistent over time, providing insight into the functional connectivity between the two signal pairs. MPC is calculated as Equation 11:

$$\text{MPC}\left(r_{1},r_{2}\right)=\frac{1}{T}\sum_{t=1}^{T}\exp\left(i\left(\theta \left(r_{1},t\right)-\theta \left(r_{2},t\right)\right)\right)\ldots$$ (11)

which directly measures the degree of phase alignment between points at $r_{1},r_{2}$ , over the time interval T (Mormann et al., 2000).

Due to the presence of beat-to-beat and topological phase synchronisation, sinus rhythm, FAT and AFL are hypothesized to have the following correlation functions, due to the periodicity of the system (Equation 12):

$$C\left(r\right)\approx 1\ldots$$ (12)

where $C\left(r\right)$ is the MPC at distance r between points, analogous to the quasi-long-range order observed in the ordered state in Kosterlitz Thouless physics.

**FIGURE 3:**
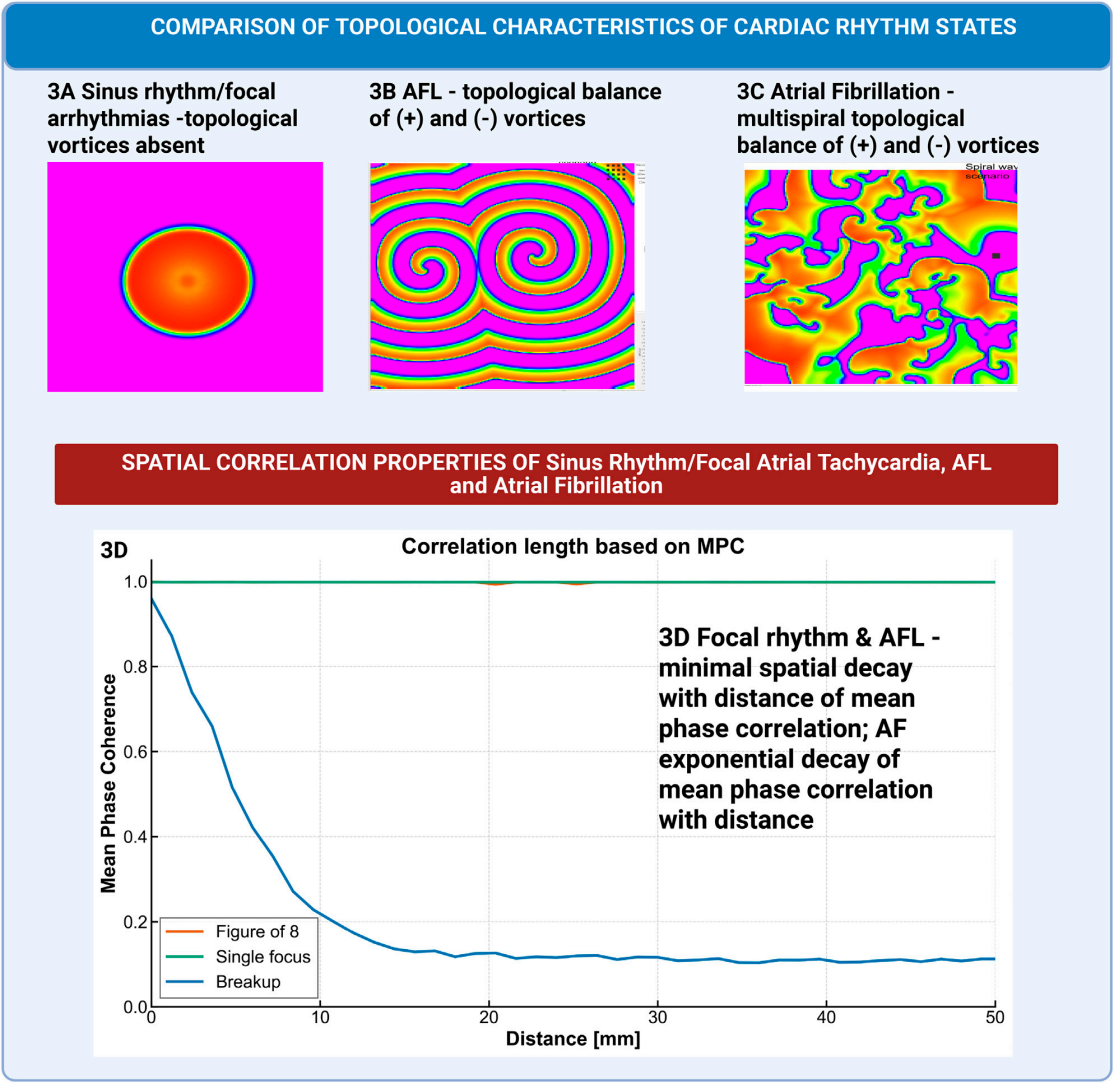
Spatial Correlation of Phase in Computational Model Different Atrial Rhythms. Mean Phase Coherence (MPC) analysis as a function of inter-electrode distance in Aliev-Panfilov computational models of different atrial rhythm states. **(A)** Focal atrial tachycardia simulation: Centrifugal activation pattern showing consistent with planar wave propagation and absence of phase singularities. **(B)** Atrial flutter simulation: Figure-of-8 reentrant pattern with paired vortices. **(C)** Atrial fibrillation simulation: Multiple spiral wave breakup showing **(D)** Quantitative analysis: Plot comparing correlation decay rates across rhythm states, with AF showing steepest decline (shortest correlation length) and focal and AFL rhythms maintaining correlation over extended distances.

In contrast, in the fibrillatory state, AF is proposed to be analogous to the disordered state in KT physics, with an exponential decay in spatial correlation (Equation 13):

$$C\left(r\right)=e^{\frac{-r}{\varepsilon }}$$ (13)

where ε is the correlation length that quantifies the typical scale over which phase coherence is maintained. An exponential decay implies that electrical activity becomes rapidly uncorrelated. In Figure 3, we show an example of spatial correlation of phase in a focal rhythm (Figure 3A), paired figure of 8 (Figure 3B), and spiral wave breakup (Figure 3C) for the Aliev-Panfilov model. Figure 3D shows spatial decay of mean phase correlation in the different states.

### Statistical properties of topological defects in the atrial fibrillatory state

The continuous regeneration of phase singularities in AF is postulated to occur via the formation and destruction of topological defects in pairs (Arno et al., 2024a; Arno et al., 2024b; Gil et al., 1990; Marcotte and Grigoriev, 2017). A consequence of this is that these events are effectively statistically independent, and hence the lifetime distribution for topological defects has been proposed to follow the following distribution (Dharmaprani et al., 2021; Quah et al., 2021; Rappel et al., 2022; Dharmaprani et al., 2022; Jenkins et al., 2022; Quah et al., 2020) (Equation 14).

$$P\left(t_{d}\right)=\lambda_{d}e^{-\lambda_{d}t_{d}}\ldots$$ (14)

where λ_d_ represents the death rate for phase singularities, t_d_ represents the singularity lifetime (Dharmaprani et al., 2019; Dharmaprani et al., 2022; Dharmaprani, 2021). Similarly, the probability distribution for inter-formation times for topological defects has been proposed to be (Equation 15):

$$P\left(t_{f}\right)=\lambda_{f}e^{-\lambda_{f}t_{f}}\ldots$$ (15)

Where λ_f_ represents the decay constant for inter-formation times, t_f_ represents the inter-formation times (Dharmaprani et al., 2019; Dharmaprani et al., 2022; Dharmaprani, 2021). By combining these probability distributions in an M/M/ ∞ birth-death process (Markovian arrival, Markovian service, infinite server) birth-death process, the statistical distribution of topological defects has been shown to follow a Poisson distribution (Dharmaprani et al., 2021; Quah et al., 2021; Dharmaprani et al., 2022; Jenkins et al., 2022; Quah et al., 2020). This is similar to other biophysical processes characterised by the regeneration of topological defects, suggesting this is a common statistical framework (Gil et al., 1990; Qiao et al., 2009; Bodenschatz et al., 2000; Ecke et al., 1995; Egolf et al., 2000) (Equation 16).

$$P_{n}=\frac{{\left(\lambda_{f}/\lambda_{d}\right)}^{n}e^{-\frac{\lambda_{f}}{\lambda d}}}{n!}$$ (16)

### Evidence supporting the characterisation of topological states of cardiac arrhythmia

Topologic considerations in sinus rhythm and focal atrial arrhythmias are relatively straightforward. These rhythms in clinical contexts are well-recognised for planar wave conduction and centrifugal activation (Callans, 2020). Topological considerations are more complex in the case of AFL and AF, for which we will provide more emphasis. We will consider each of these cases separately and in more detail.

### Topological considerations in AFL

AFL is the archetypal macro-re-entrant arrhythmia of the atrium (Callans, 2020; Saoudi et al., 2001). Atrial flutters are characterised by large coherent wavefronts, in contrast to the relatively disorganised electrical activity observed in AF. Macro-re-entry in the clinical literature is believed to occur as a consequence of slowing in wavefront conduction, anatomic barriers, or both, and is classically described as re-entry around a single large obstacle (Boineau et al., 1980). On the surface ECG, AFL is typically characterised by continuous undulation of atrial electrical activity between QRS complexes that arise from the ventricle.

A characteristic of AFL is that the arrhythmia circuit can be entrained (Waldo et al., 1977). Entrainment is a manoeuvre in which the arrhythmia is paced at a cycle length (CL) slightly faster than the original CL of the tachycardia. At sites considered within the circuit, entrainment results in a return cycle at or only slightly longer than the tachycardia cycle length. In contrast, at sites outside the tachycardia cycle length, entrainment leads to a return cycle much longer than the CL of the tachycardia.

In its typical form, which accounts for 90% of AFLs, atrial activation proceeds counterclockwise in myocardium around the tricuspid annulus (Callans, 2020; Saoudi et al., 2001; Issa et al., 2009). The typical anatomical boundary of the circuit is the tricuspid valve annulus anteriorly, with the posterior boundary formed by the crista terminalis where a line of double potentials is typically observed. In clinical descriptions of the typical AFL circuit, wavefront propagation is described as preceding cranio-caudally on the lateral wall of the right atrium, and caudo-cranially in the right atrial septum. AFL is conventionally described as a single circuit, with the location of successful ablation (∼effective in >95% of cases) at the cavo-tricuspid isthmus which is located between the tricuspid valve annulus and the inferior vena cava. Although AFL is typically described as a single circuit, with sites considered within the circuit defined by entrainment.

A clinically important variant is lower-loop re-entry (Cheng et al., 1999), in which a second circuit is recognised around the inferior vena cava (usually with a clockwise orientation when viewed from below). This is not considered in general in the clinical literature as a mandatory phenomenon, and in classic clinical descriptions is considered as a variant with figure-of-8 type morphology in which entrainment can occur from this second loop. Notably, the successful ablation location is at the intersection of the two loops in the cavotricuspid isthmus.

In contrast to typical AFL, where the anatomical circuit is considered well-defined in the right atrium, in atypical AFL (which comprises 10% of flutters), atypical AFL can involve circuits in either the left or right atrium (Kalman et al., 1997). Typical clinical associations for atypical AFL may include previous cardiac surgery, particularly for the correction of congenital heart disease, or prior catheter ablation. These are believed to create scars and areas of relatively slowed conduction.

In contrast to typical AFL, identification of the critical targets for ablation in atypical AFL may be difficult. Multiple potential circuits and morphologies are possible, with anchoring of re-entry possible to previous scars or anatomical boundaries (Johner et al., 2025; Jaïs et al., 2000). In contemporary clinical practice, the approach to mapping these circuits commonly consists of annotation of local electrical activation in an electroanatomic mapping system, in combination with the utilisation of entrainment to facilitate localisation of the circuit (Johner et al., 2025). Despite the combination of these approaches, mapping of atypical AFL may be challenging, with procedures often taking more than 2 h. Atypical AFL ablationi may also have relatively high rates of complications and recurrence (Pott et al., 2022; Barradas et al., 2023; Raymond-Paquin et al., 2023).

A key problem in mapping of atypical AFL is the identification of the key location for ablation (Johner et al., 2025). Although clinical studies have provided important empirical insights into the approach to ablation via activation mapping and entrainment approaches, at present with these approaches it may be difficult to identify or treat the critical zone anchoring the tachycardia, which has led to the search for a new mechanistic framework to understand AFL.

### New clinical evidence for a unified topological framework for AFL

**FIGURE 4:**
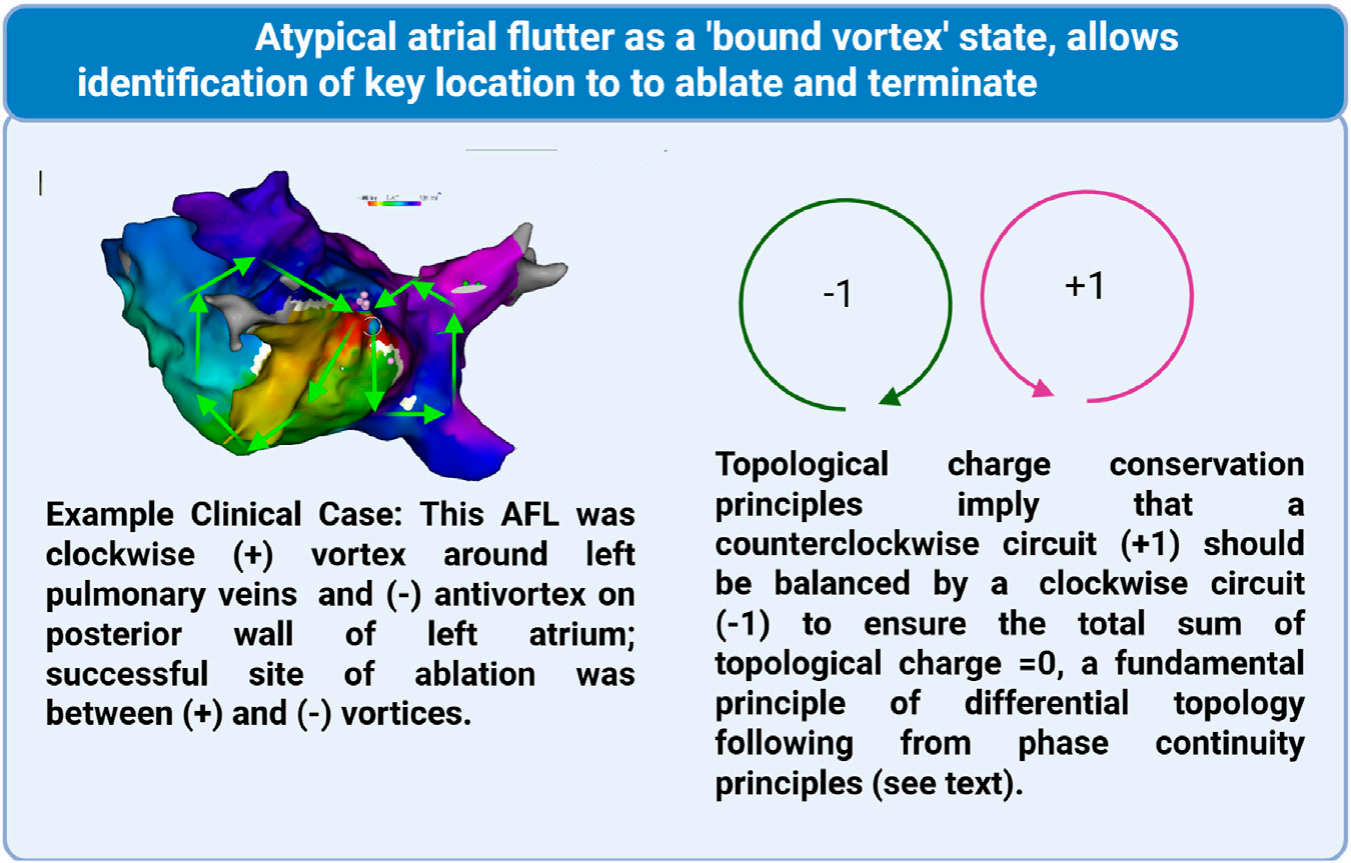
The Paired Loop Paradigm in Atrial Flutter. Illustration of the topological model for Atrial Flutter (AFL), demonstrating the concept of paired critical boundaries (CBs) with opposite topological charges (+1 and −1). **(A)** Symmetrical dual-loop reentry around two CBs, in a clinical case showing the pairing of loops in a local activation map from a patient with atypical AFL. **(B)** Schematic of topological loop pairing.

In this context, exciting new evidence has emerged that AFL may be considered as a topologically balanced circuit with positive and negatively chiral vortices (Vandersickel et al., 2024; Vandersickel et al., 2023) creating quasi-long-range order. In hybrid studies combining theoretical computational modelling and retrospective investigation of clinical atypical AFL cases, Vandersickel and co-workers have recently demonstrated that all cases of human AFL can be conceptualised as positive and negatively chiral vortices, bound by a common isthmus (Vandersickel et al., 2024; Vandersickel et al., 2023). A schematic of this concept is illustrated in Figure 4.

The essential concept proposed by Vandersickel is that AFL can be conceptualised as positive and negatively chiral vortices, occurring as loops of wave propagation around anatomical or scar boundaries. Topological balance occurs because the chirality of loops with opposite topological charge (+1 and −1) in the phase field balance, and hence sum to zero (Vandersickel et al., 2024; Vandersickel et al., 2023). Their existence as pairs is fundamentally linked to the continuity of this phase field across the atrial surface and the principle of topological balance; within a defined region, the net topological charge must often remain constant (typically zero), meaning the creation of a singularity with one charge necessitates the simultaneous creation of another with the opposite charge nearby to maintain this overall balance, analogous to charge conservation.

This contrasts with the paired vortices found in the low-temperature bound state of Kosterlitz-Thouless (KT) physics, which describes phase transitions in 2D systems. In KT physics, vortices are also topological defects with opposite circulations (+1 and −1), but their pairing at low temperatures is primarily an energetic phenomenon. In contrast, in AFL setting pairing of +1 and −1 vortices occurs as a consequence of wave dynamics and substrate geometry, due to the mathematical necessity of conserving topological charge within the continuous phase field.

These studies introduce a unifying framework for understanding including typical and atypical AFL, based on atrial topology. This approach models the atrium as a closed surface with a finite number of non-conductive boundaries (holes), such as valves, veins, or scar tissue. According to the index theorem, the sum of topological charges (representing wave rotation around boundaries) must be zero (Vandersickel et al., 2024; Vandersickel et al., 2023). This mathematical principle leads to the “paired loop paradigm,” which dictates that re-entrant circuits in AFL and atypical AFL must always involve an even number of critical boundaries (CBs), specifically pairs rotating in opposite directions (one clockwise, TC = −1; one counterclockwise, TC = +1). This is in line with the long-standing principle of topological charge preservation.

This construct explains why, in atria with three or more boundaries (e.g., left atrium with mitral valve, left veins, right veins), atypical AFL consistently manifests as dual-loop activation around two CBs, with parallel activation around any remaining non-critical boundaries (NCBs). A crucial observation that is often overlooked in clinical mapping, is that these paired loops are often asymmetrical, meaning one loop might complete a full rotation (“true loop”) while the second is nearly complete but appears “suppressed” or passive due to collision with the first wave (Vandersickel et al., 2024; Vandersickel et al., 2023). Despite potentially showing long post-pacing intervals (dPPI) characteristic of passive tissue during entrainment mapping, this second loop (CB2) is equally critical according to the topology construct (Vandersickel et al., 2024; Vandersickel et al., 2023).

Traditional interpretations of AFL have typically focused only on the dominant loop (CB1), essentially treating the flutter circuit as a “leading circle” phenomenon. The problem with this approach is that by underestimating the importance of this second, paired loop, an incorrect ablation target is selected, leading the flutter circuit to shift and activating previously non-conducting boundaries.

The clinical significance lies in predicting ablation outcomes and guiding strategy. The construct predicts that terminating the atypical AFL requires ablating the connection between the *two* critical boundaries (CB-CB ablation), regardless of asymmetry. Ablating only the dominant loop by connecting it to a non-critical boundary (CB1-NCB ablation) in an asymmetrical case will not terminate the tachycardia but will instead often lead to it slowing down as the previously suppressed second loop (CB2) takes over, now rotating in an N-1 boundary configuration (Vandersickel et al., 2024; Vandersickel et al., 2023). Understanding this paired-loop dynamic provides a unified explanation for various activation patterns, entrainment results (including long dPPIs at critical sites), and why some ablations terminate flutter while others only slow it down, potentially simplifying diagnosis and improving ablation success rates (Vandersickel et al., 2024; Vandersickel et al., 2023).

Clinical validation by Duytschaever and Vandersickel involved analyzing 131 atypical and typical AFL cases (106 in the left atrium [LA], 25 in the right atrium [RA]). In the LA, flutter predominantly occurred in 2-boundary (often the mitral valve [MV] paired with combined pulmonary veins [PVs] post-roof line) and 3-boundary topologies (typically involving MV, left PVs, and right PVs as boundaries, with common pairings like MV-LPV, MV-RPV, or LPV-RPV as the two CBs) (Vandersickel et al., 2024). In the RA, a 3-boundary setup (tricuspid valve [TV], inferior vena cava [IVC], superior vena cava [SVC]) was most common, usually resulting in paired loops around the TV and IVC as CBs (Vandersickel et al., 2024). Scar tissue or prior surgical lines could act as additional boundaries, creating 4-boundary scenarios in either atrium. The observed ablation outcomes aligned with the construct: 75% terminated upon connecting the two identified CBs, while 18% converted to a slower tachycardia when a dominant CB was connected to an NCB (Vandersickel et al., 2024), demonstrating the clinical relevance of the paired, often asymmetrical, loops.

Further clinical support comes from Santucci et al., who, through detailed electroanatomical mapping of 128 atypical flutter cases (100 LA, 28 RA), independently derived a similar core finding (Santucci et al., 2024). They proposed a method to classify conduction barriers based purely on activation patterns, defining “critical boundaries” (CBs) as those showing sequential activation spanning ≥90% of the tachycardia cycle length. In every case, they identified exactly two such distinct CBs. Ablation creating a line connecting these two empirically identified CBs terminated the tachycardia in 96% of cases where block was achieved (Santucci et al., 2024). While the clinical outcome and the central observation – the necessity of identifying and connecting two critical boundaries for successful ablation – are identical to the conclusions drawn from the topology/index theorem framework, Santucci’s approach was derived empirically from mapping criteria without explicitly referencing or utilizing the underlying mathematical or topological concepts, the demonstration of near identical results provides clinical validation of this novel paradigm (Santucci et al., 2024).

### AF as an unbound vortex state

Although the mechanisms of clinical fibrillation are considered incompletely resolved at the current time (Roney et al., 2020; Jalife, 2011; Handa et al., 2020; Schotten et al., 2011; Moe, 1962; Moe and Abildskov, 1959; Moe et al., 1964), a significant body of evidence exists consistent with the notion that many and possibly the majority of AF patients suffer from a form of scroll wave turbulence (Biktashev, 1998; Winfree, 1994). Scroll wave turbulence may be considered the 3-Dimensional comparator disordered dynamics that can arise from spiral wave breakup in 2-Dimensional model systems (Karma, 1994; Kuklik et al., 2010; Panfilov, 1998; Ten Tusscher and Panfilov, 2003; Ten Tusscher and Panfilov, 2006; Yang et al., 2003; Fenton et al., 2002). In 2D, instabilities may cause spiral waves to “break up” into multiple, disorganised spirals causing highly complex, spatiotemporally disordered patterns of activation.

The key characteristic of scroll-wave turbulence is repetitive creation and annihilation of phase singularities that are organised in 3-Dimensional filaments. The topological construct of scroll wave turbulence has the potential to account for a number of the consistent clinical observations that have been noted in the field. These include: (i) electrical and mechanical disorder, causing changing local activation characterised by unstable re-entrant circuits; (ii) spatiotemporal monotonic exponential decay with distance of local coherence of locally observed clinical electrograms is a consistent observation in AF. (Dharmaprani et al., 2024; Botteron and Smith, 1995; Botteron and Smith, 1996); (iii) dissociation of electrical activity on the endocardial and epicardial surfaces of the atrium (Allessie et al., 2010; de Groot et al., 2016) (iv) difficulties in identifying specific source regions driving clinical AF that would be amendable to localised focal treatment such as catheter ablation.

The statistical properties of topological defects observable in atrial fibrillation are consistent with the expectations of the scroll wave turbulence. Due to the pairwise formation and annihilation processes (Arno et al., 2024a; Winfree, 1989; Coullet et al., 1989; Gil et al., 1990; Lega, 1991), it would be anticipated that topological defects in fibrillation would have exponential lifetime distribution, exponential-inter-formation time distributions, and a Poisson distribution of population defects (Dharmaprani et al., 2019; Dharmaprani et al., 2022; Jenkins et al., 2023; Jenkins et al., 2022; Dharmaprani, 2021). These properties are consistent with the statistical characteristics of topological defects in other turbulent systems in nature (Qiao et al., 2009; Bodenschatz et al., 2000; Ecke et al., 1995; Coullet et al., 1989; Gil et al., 1990; Lega, 1991; Morris et al., 1993; Ecke and Hu, 1997).

In this regard, there are certain similarities of scroll wave turbulence to the high-temperature (disordered) phase of KT systems, where topological defects (like vortex-antivortex pairs) are unbound and proliferate freely. In the high-temperature KT phase, the system is also disordered, populated by free, unbound defects. While originating from an equilibrium framework, the motion and encounters of these free defects lead to annihilation events when oppositely charged defects meet. Although less commonly the primary focus, compared to the phase transition itself, the random nature of these encounters in the dense, disordered phase is also leads to approximately exponential distributions for defect lifetimes, similar to scroll wave turbulence. The key factor is the relative absence of long-range binding forces dominating the dynamics.

**FIGURE 5:**
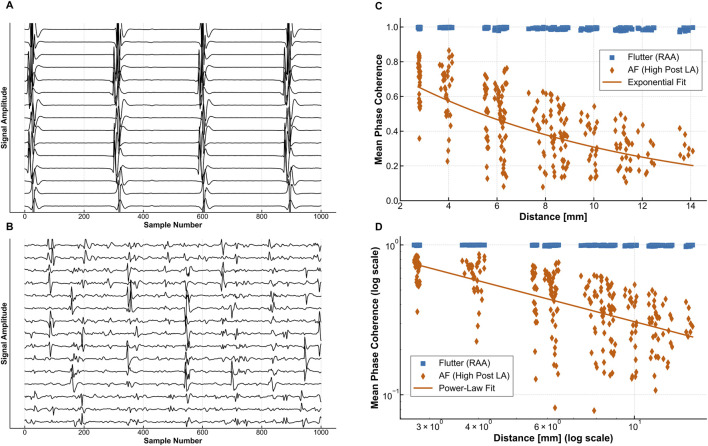
Comparison of Spatial Correlation Properteis in Human AFL and AF. Improved Caption: “High-density grid electrode recordings demonstrating distinct spatial correlation patterns in human atrial arrhythmias. **(A)** AFL electrograms: Representative 10-s recordings from a 64-electrode grid showing organized, periodic activity with consistent cycle length (CL ≈ 250 ms) and stable morphology across adjacent electrodes, indicative of macro-reentrant activation. **(B)** Atrial fibrillation electrograms: Corresponding recordings from the same electrode array showing irregular, polymorphic activity with variable cycle lengths (180–400 ms) and rapid morphology changes, characteristic of multiple wavelet dynamics. **(C)** Mean Phase Coherence analysis: Quantitative comparison showing MPC decay as a function of inter-electrode distance. AFL maintains high coherence (MPC>0.7) even at distances >40mm, consistent with quasi-long-range order, while AF shows rapid exponential decay (MPC<0.3 at 20 mm distance), confirming short correlation length ε ≈ 8–12 mm. **(D)** Mean Phase Coherence Plot: Demonstrates exponential decay in AF absence of decay in AFL providing clinical validation of the topological state hypothesis with AF resembling the KT unbound phase and AFL resembling the bound phase.

Regarding spatial coherence, the similarity provides an important analogy. Scroll wave turbulence, being inherently chaotic and disordered, exhibits spatial correlations that decay exponentially with distance, characterized by a finite correlation length. This signifies the absence of long-range order. Similarly, in the high-temperature phase of a KT system, the presence of numerous free, unbound defects effectively screens the interactions, destroying the quasi-long-range order found below the transition. Consequently, spatial correlation functions in this phase also decay exponentially with distance (Cheung, 2011; Nishimori and Ortiz, 2011). An example of clinical electrogram data is shown in Figure 5, showing differences in spatial correlation as determined by Mean Phase Coherence in the AFL and AF states.

An additional feature of KT physics that may be relevant is the role of system spatial size considerations as a determinant of dynamics. In the XY model, the concept of configurational entropy relates to the interaction of these defects and the number of potential spots (independent regions) for defects to occupy. The L/a ratio becomes crucial in this context, as it effectively determines the landscape on which these singularities can form and interact. When the L/a ratio increases, in line with a Peirls’ type argument, the relative configurational entropy increases, and increases the relatively stability of the disordered unbound vortex state (Cheung, 2011; Nishimori and Ortiz, 2011).

Similar scaling properties may also be relevant in the context of fibrillation. The scaling properties associated with self-organisation of fibrillatory transients have been systematically studied (Dix et al., 2024; Lilienkamp et al., 2017; Lilienkamp and Parlitz, 2018a; Lilienkamp and Parlitz, 2018b). The average duration of multispiral transients scales exponentially with system area (Dix et al., 2024; Lilienkamp et al., 2017; Lilienkamp and Parlitz, 2018a; Lilienkamp and Parlitz, 2018b), and compares to supertransients in coupled map lattices (Kaneko, 1985; Kaneko, 1990; Stahlke and Wackerbauer, 2009). Similar scaling properties have been identified in human AF. The number of independent regions, estimated by the ratio of system size to correlation length L/ξ (Dharmaprani et al., 2024), was shown to have a power-law relationship with observable fibrillation episode durations. Essentially, this translates to the idea that the larger the number of independent regions, the more complex the interactions of defects, and therefore making it less likely to transition from out of the unbound vortex fibrillatory state. This concept aligns with the notion of the critical mass hypothesis (Kim et al., 1997; Zipes et al., 1975), in which system size is a key determinant of the stability of the fibrillatory process (Hartley et al., 2021). In the context of topological consideration of fibrillation, increased system size potentially is considered to lead to greater configurational entropy and increases the relative stability of fibrillatory of the process.

A topological model of AF also provides a potential framework for understanding the common clinical observation that catheter ablation for AF, particularly pulmonary vein isolation (PVI), can lead to the development of organised AFL. From a topological perspective, the lines of ablation created during PV*I* introduce new, enlarged, non-conducting boundaries within the atrial tissue. This alteration of the atrial electrophysiological properties can fundamentally change the dynamics of topological defects. In a state of AF, conceptualised as an unbound vortex state with chaotic, fleeting vortices, the creation of large, stable boundaries may facilitate the anchoring of previously transient re-entrant circuits. By providing a structure for vortices to attach to, the ablation lines can induce a transition from the disordered, unbound state (AF) to a more stable, bound vortex state (AFL), where paired vortices of opposite chirality are anchored to these new anatomical constraints. This may facilitate new macro-re-entrant tachycardia, while more organized, is also more stable; however, the topological framework predicts that this new circuit is dependent on the connection between these boundaries, offering a clear and rational target for subsequent curative ablation. Such a concept may provide a pathway to reconcile results of recent clinical trials where improvements in AF reduction have typically been met by compensatory increases in atypical AFL outcomes (Deisenhofer et al., 2025).

### Facilitating transitions between AF and AFL states, comparison to KT physics

A fundamental distinction between KT transitions and AF and the AFL states lies in the nature of the driving forces. The Kosterlitz-Thouless transition is rooted in equilibrium statistical mechanics. The unbinding of topological defect pairs (like vortex-antivortex pairs) is driven by thermal fluctuations. As the system’s temperature increases towards the critical point, the thermal energy becomes sufficient to overcome the logarithmic binding potential energy holding the pairs together. This process is governed by the system minimizing its free energy, representing a transition between thermodynamic equilibrium states–one with bound pairs (quasi-long-range order) and one with free topological defects.

In contrast, the transition from a more organized state like AFL to the turbulent state of AF is a non-equilibrium biological process driven by electrophysiological instabilities rather than thermal equilibrium. While the end state (AF) resembles a defect-rich turbulent phase, the creation of these defects (spiral/scroll wave tips via wavebreaks) is not primarily due to unbinding of pre-existing pairs driven by thermal-like energy. Instead, it may be potentially triggered by specific arrhythmogenic events such as stochastic fluctuations in ion channel activity leading to early or delayed afterdepolarizations (EADs/DADs) (Vandersickel et al., 2014), the interaction of wavefronts with tissue heterogeneities (like scar tissue or fibrosis) (Dix et al., 2024; Shajahan et al., 2007; Shajahan et al., 2009), structural remodelling altering tissue conductivity and size, or dynamic instabilities related to wave curvature and restitution properties, especially in the context of an enlarged or diseased atrium (altered system size and substrate). These factors introduce perturbations that cause wavebreaks and initiate the disordered cascade characteristic of AF.

## Discussion

### A topological phase model of a cardiac rhythm state

In this perspective paper, we propose a hypothesis of considering different cardiac atrial rhythm states as alternative topological states, and thereby the transitions between these states as a form of nonequilibrium phases transitions. The concept is rooted in the theoretical principle of topological charge conservation, which is logical conceptual paradigm that has extensive theoretical support for several decades. The of this hypothesis are that:

- Sinus rhythm and FAT are considered as states characterised by the absence of phase singularities, with topological charge = 0 at all points of the atrial chambers, and relative spatial long-range order.
- AFL is characterised by paired vortices of opposite topological charge (−/+1), with quasi-long-range order maintained by this topological balance.
- AF is characterised by the repetitive regeneration +1 and −1 topological charged defects, account for observed disorder, but maintaining overall topological charge balance.

### Utility of a topological model of cardiac rhythm state

A topological framework offers a potentially useful lens for understanding cardiac rhythm states and their transitions, drawing parallels with topological phase transitions in physics. As outlined earlier, a key characteristic proposed for the cardiac phase field is the conservation of topological charge (summing to zero). This constraint inherently limits the possible stable configurations, potentially defining discrete rhythm states. We hypothesize that SR & AF, AFL, and AF represent such distinct topological states, analogous to the distinct phases observed in Kosterlitz-Thouless (KT) physics.

Several comparisons arise between these proposed atrial topological states and KT phases. It is observed that AFL is characterized by stable, paired topological vortices, analogous to the KT bound vortex state which establishes long-range order. Conversely, we propose that AF, often described as scroll wave turbulence, resembles certain aspects oof the KT unbound vortex state. This state is associated with topological disorder and the characteristic exponential decay of spatial correlations observed in AF.

However, a crucial distinction exists between the proposed cardiac model and KT physics regarding the *mechanism* driving these states. In KT systems, phase transitions arise from free energy considerations governing vortex binding. In the cardiac context, we propose the different states emerge primarily from the mathematical necessity of maintaining phase field continuity, mandating zero net topological charge across the tissue. While energy considerations likely play a role in the *stability* and *transitions* between cardiac states, the fundamental *existence* of these discrete states may stem from this topological constraint.

Exploring the potential utility of such discrete topological states in cardiac electrophysiology is intriguing. A hallmark of topological phases in physical systems is their robustness against minor perturbations; a significant energy barrier (related to vortex unbinding/binding) must be overcome to induce a phase transition. This aligns with the fundamental property of topologically ordered excitable media, where small, sub-threshold perturbations are rapidly “damped out”. We speculate that the topological order inherent in the sinus rhythm state (characterized by the absence of vortices) contributes significantly to this damping effect and robustness. Minor electrical disturbances, such as premature atrial contractions or transient localised conduction delay or block, represent small local perturbations to the global phase field. The requirement to maintain the overall topological state (zero net charge, no vortices) could act as a stabilizing factor, effectively damping these perturbations and preventing them from triggering the large-scale reconfiguration (i.e., vortex creation/unbinding) necessary to transition into AFL or AF. In essence, the topological integrity of sinus rhythm imposes a threshold that minor disturbances fail to cross.

This inherent stability against frequent, minor electrophysiological perturbations secondary to ectopic rhythms offers a potential evolutionary advantage. The ability of the heart to consistently maintain sinus rhythm, despite constant physiological fluctuations, is crucial for ensuring reliable circulatory function. The topological protection of the sinus state could therefore be an evolutionarily selected feature, providing a robust baseline rhythm, maintaining coordinated cardiac contraction, and reducing the organism’s propensity towards sustained, potentially lethal arrhythmias, thereby enhancing survival fitness. Further investigation is needed to explore whether this proposed topological stability quantitatively accounts for the heart’s remarkable resilience to arrhythmogenesis.

### Clinical implications of a topological model of a cardiac rhythm state

A topological construct as outlined above has immediate potential clinical utility for the mapping of FAT and AFL. In the context of FAT, mapping of the primary arrhythmia as a focal arrhythmia by the absence of complete circuits at any point in the atrium (i.e., topological charge 0), particularly around non-conducting regions such as the valve, and vein origins, and local scar. Such a mapping concept, which would be easy to identify with modern electroanatomic mapping systems, would enable identification of FAT without perturbing the arrhythmia with extrastimuli as required by current clinical approaches such as ventricular entrainment, or programmed atrial extrastimuli.

For AFL, both typical and atypical, the topological approach has immediate therapeutic implications. The notion of targeting the region between positively and negatively chiral vortices leading to simultaneous collapse with preservation of topological charge is new and will immediately augment established clinical approaches of activation mapping and entrainment. The approach has the advantage of mechanistic and theoretical underpinning, allowing the interpretation of complex activation maps to potentially be made easier allowing identification of the critical zone for ablation, thus forming a construct of practical assistance to electrophysiologists and patients.

In the context of AF, the topological model could support a scenario wherein AF is sustained by repetitive regeneration of vortices. A topological model of AF and AFL could allow for improved understanding of the transition between AF and AFL, which is observed commonly clinically, for example, during sodium-channel blockade (Kneller et al., 2005). Detailed characterisation of the topological properties of phase singularities in human AF is underway (Quah et al., 2020). A potentially testable prediction is that transitional states in AF would show spatiotemporal intermittency (Kaneko, 1985; Zaritski et al., 2004; Chaté and Manneville, 1988; Chaté and Manneville, 1994; Ciliberto and Bigazzi, 1988; Daviaud et al., 1990; Kaneko, 1990), due to a power-law distribution of laminar and turbulent domains during the fibrillatory state. Assessing the birth and death of topological defects in human AF (Rappel et al., 2022), which has previously been validated, could potentially be complementary to alternative efforts to search for driving spiral waves in AF (Rappel et al., 2024).

An additional potentially important consideration that could arise from the principle of topological charge conservation is in relation to the role of anatomical boundaries. An important implication of the principle of topological charge conservation is that wavefronts can attach to anatomical boundaries and the circulation of wavefronts around those boundaries must contribute to topological charge considerations at the whole chamber level. This is relevant in that large lesions, or complex structured lesions such as wide antral circumferential ablation, posterior wall isolation, or even the recently postulated ablation of regions of spatiotemporal dispersion, may lead to anchoring of wavefronts, and hence a secondary propensity to macro-re-entrant tachycardia. This hypothesis may underly the interdependence between outcomes of AF recurrence and macro-re-entrant atypical AFL in contemporary AF ablation randomised trials (Deisenhofer et al., 2025; Kistler et al., 2023; Cao et al., 2025). The detection of systematic anchoring to regions of ablation is a further hypothesis that could be tested during AF ablation studies, to understand the biophysical processes by which AF ablation facilitates AF termination.

### Addressing unknowns and future directions

While a topological construct for atrial arrhythmia holds promise, several unknowns must be addressed. The atrium operates as a nonequilibrium system, and its interaction with other aspects of AF biology, such as fibrosis, requires detailed further investigation. The investigation of topological properties in clinical investigations will need to be carried out in detail. Prospective studies applied to AFL mapping will need to be performed to validate the conceptual paradigm proposed here. New tools and technologies facilitating high-density mapping of the atrium may facilitate detailed exploration of AF (Yavin et al., 2021). A key challenge if a topological model of AF is supported, is how to use this information to derive physics-based ablation strategies. There is an urgent need in the clinical community for objective, explainable approaches to catheter ablation of AF (Kistler and Chieng, 2019), and a topology-based approach could offer a means to understand and determine ablation strategy. We caution that the purpose of this contribution is to be a potential hypothesis to motivate future investigations, rather than definitive proof that a topological approach will lead to direct clinical benefit.

## Conclusion

In summary, this hypothesis article proposes for a topological consideration AF, AFL and FAT, inspired by Kosterlitz-Thouless physics. By viewing AF as an unbound vortex state and AFL as a bound vortex state, we can gain deeper insights into the mechanisms driving these arrhythmias. This approach offers a promising avenue for developing new treatments and improving clinical outcomes for patients with AF and AFL. Through collaborative research efforts, we can further elucidate the complex dynamics of these arrhythmias and enhance our ability to manage them effectively.

## Data Availability

The original contributions presented in the study are included in the article/Supplementary Material, further inquiries can be directed to the corresponding author.
