# Supplementary material for: A topological hypothesis for atrial fibrilllation, atrial flutter and focal atrial tachycardia: comparison and contrast with Kosterlitz-Thouless physics
Source: Front Netw Physiol. 2026 Jan 8;5:1710567. doi: 10.3389/fnetp.2025.1710567 (PMC12823853; doi:10.3389/fnetp.2025.1710567)
Supplement: Supplementary file 1 [file DataSheet1.docx]

SUPPLEMENTAL INFORMATION

The following section has methodological information on the implementation of electrophysiological models, and calculation of correlation data used in this manuscript.

Part 1 Electrophysiological Model Details

#### Electrophysiological Model

To investigate the correlation length decay of electrograms in cardiac tissue during fibrillation and flutter. we employed the ten Tusscher Panfilov (TTP) model [R1]. The **model** is a **simplified two-variable reaction-diffusion model** that captures the essential features of **excitable cardiac tissue** while remaining computationally efficient. The model distills the fundamental dynamics of action potential propagation into a minimalistic framework. This makes it particularly well-suited for large-scale simulations of atrial dynamics, where the primary interest is in **wave propagation, reentry patterns, and spiral wave dynamics**, rather than precise ionic mechanisms. In the context of **atrial fibrillation and flutter**, where the key phenomena involve the breakup of wavefronts and the formation of stable or unstable reentrant circuits, the TTP model provides a computationally efficient approach to studying the effects of tissue heterogeneity, fibrosis, and conduction anisotropy on arrhythmogenesis. While it does not explicitly model individual ion channels, its phenomenological representation of excitation and recovery captures the essential qualitative behavior of atrial action potentials, making it a valuable tool for understanding macroscopic electrical activity in the atria.

#### Numerical Implementation

The simulations were performed on a two-dimensional tissue domain using a monodomain formulation with t**ransmembrane voltage defined as:**


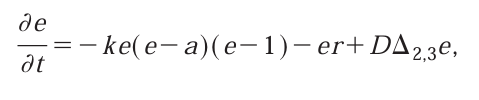
where, *e* represents the transmembrane voltage, *D* is the diffusion coefficient reflecting the electrical coupling between cells, *k* and *a* are parameters controlling the shape of the action potential, Δ is Laplacian operator and *r* is a conductance of slow inward current defined as:

The parameters of the model do not have a clear physiological meaning but are adjusted to reproduce key characteristics of cardiac tissue, such as the shape of the action potential, refractoriness and restitution of action potential duration. The values used in this study are: a=0.1, m2=0.3, k=8, e=0.01, b=0.1, while parameter m1 was set to 0.2 to produce stable spiral waves scenario and 0.05 to produce spiral wave breakup. Simulations were conducted on 200x200 two-dimensional grid with dx=0.2 and dt=0.02 using Forward Euler scheme.
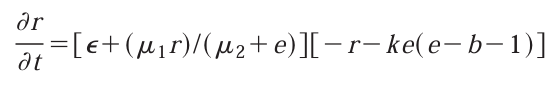
Simulations were conducted for 300 t.u. (time units of the model) to obtain at least 30 cycles in case of spiral wave rotation. In case of spiral wave break simulations were conducted for 1300 t.u. to obtain 100 cycles.

#### Part 2 Analysis of electrogram data

In **KT theory**, correlation length is calculated based on the orientation of lattice spins. In cardiac tissue, we do not deal with spins but rather with **propagating waves**. Therefore, we estimated **correlation length** based on a measure of **electrogram phase coherence** between two spatial points in the system. The phase of the electrogram was obtained using **sinusoidal recomposition** [R2], followed by the **Hilbert transform**. The phase ϕ(t) of the electrogram was defined as:

$\phi(t)=\text{arctan}\left( \frac{-u(t)}{H(u)(t)} \right)$

where u(t) is the **recomposed electrogram**, and H[u(t)] is the **Hilbert-transformed electrogram**.

Electrogram coherence was quantified using the concept of **Mean Phase Coherence (MPC)** [R3]. MPC was calculated based on **electrogram phase values across all time points**. The correlation between electrograms at spatial points r1 and r2 is defined as:

$\text{MPC}(r_{1},r_{2})=\frac{1}{T}\sum_{t=1}^{T} \text{exp}(i(\phi(r_{1},t)-\phi_{k}(r_{2},t)))$

where *t* **denotes the time sample index**, and ***T* is the total number of time samples in the electrogram**.

The **correlation length curve** for a given simulation was obtained by randomly selecting **10,000 pairs of spatial points** and calculating MPC for the corresponding electrograms. A **moving average filter** with a bin size of **1 mm** was then applied to obtain the correlation curve.

[R1] K. H. W. J. ten Tusscher and A. V. Panfilov, Influence of nonexcitable cells on spiral breakup in two-dimensional and three-dimensional excitable media, PHYSICAL REVIEW E 68, 062902 2003

[R2] Kuklik, P., et al., Reconstruction of instantaneous phase of unipolar atrial contact electrogram using a concept of sinusoidal recomposition and Hilbert transform. IEEE Trans Biomed Eng, 2014.

[R3] Mormann, F., et al., Mean phase coherence as a measure for phase synchronization and its application to the EEG of epilepsy patients. Physica D, 2000. 144(3-4): p. 358-369.

Part 3 – Example Code

Example code to generate XY model snapshots in Figure 2 of the paper is provided in the Supplement File ‘XY model- generate model snapshots.py’.

Example code for ten Tusscher Panfilov model provided in TP1A_2D_system.cpp; TP1A_2D_system.h

Example code for analysis of human electrogram signals is provided in the Supplemental File HD Grid analysis code v.1.0.py.
